# Perceptions of Stress of Swedish Volunteer Youth Soccer Coaches

**DOI:** 10.3390/sports8110146

**Published:** 2020-11-03

**Authors:** Krister Hertting, Stefan Wagnsson, Karin Grahn

**Affiliations:** 1 School of Health and Welfare, Halmstad University, S-30118 Halmstad, Sweden; 2 Department of Educational Studies, Karlstad University, S-65188 Karlstad, Sweden; stefan.wagnsson@kau.se; 3 Department of Food and Nutrition and Sport Science, University of Gothenburg, S-40530 Gothenburg, Sweden; karin.grahn@ped.gu.se

**Keywords:** coaching, soccer, youth sport, voluntary coaches, stress

## Abstract

Background: The work of a coach can be stressful, and little is known about how volunteer coaches in child and youth soccer perceive stress. Therefore, the overall aim of this study was to explore perceptions of stress among Swedish volunteer youth soccer coaches. Methods: An online questionnaire was distributed to 1514 soccer coaches of which 688 (78% men and 22% women; 4% < 30 years, 34% 31–40 years; 57% 41–50 years and 5% > 51 years) with non-profit positions responded. Results: Findings indicate that participants in general do not feel excessively stressed by being a volunteer youth soccer coach (M = 2.20; SD = 0.93; Min = 1; Max = 5), and no significant differences in perceived stress level were found based on gender, age, ethnicity, educational level or occupation. Multiple regression analysis showed that demands from employment (β = 0.24, *p* < 0.001), difficulty catching up with the family (β = 0.22, *p* < 0.001), not having enough time to plan activities (β = 0.13, *p* < 0.001), feeling pressured when selecting the team (β = 0.09, *p* = 0.013) and own demands to achieve good results (β = 0.07, *p* = 0.045), significantly contributed to perceptions of stress among the investigated youth sport coaches. Conclusions: The results shed light on the important aim that sport clubs develop holistic strategies when recruiting and retaining coaches and for other functions concerning child and youth soccer teams.

## 1. Introduction

Being a coach for children and youth teams can be stressful and negatively affect the coaching and learning environment with the youngsters. Surujlal and Nguyen [1] argue that the health and wellbeing of coaches is a necessity in educating and inspiring others. In addition, Stebbings et al. [2] stress that coaches who experience higher levels of positive affect are more likely to trust their athletes’ abilities and encourage empowering possibilities for these athletes. This is supported by Alcaraz et al. [3] and Thellwell et al. [4] who suggest that coaches who experience psychological well-being are more likely develop healthy relationships with athletes.

In Sweden and the Scandinavian countries there is a strong emphasis on club sport voluntary coaching, and in the European Commission’s Eurobarometer on sport and physical activity 25% of Swedish respondents indicated that they volunteered for sports clubs [5,6]. There are approximately 23,000 sports clubs in Sweden, which are mainly based on children’s and youth sports, and in 2014 almost 70% of Swedish children aged 7–14 years were engaged in a sports club [7].

Norris et al. [8] maintain that there is a lack of research on stress and psychological well-being in sports coaches at different competitive levels. Research is mostly conducted on high performance coaching and has predominantly focused on burn out [9,10,11,12,13]. In a critical review of psychological literature on stress among sport coaches, Fletcher and Scott [14] found that in general coaches at higher levels of competitive sports encounter greater numbers of stressors compared to lower level coaches. However, Fletcher and Scott [14] underline the need to further consider personal and situational characteristics in coaches’ perceptions of stress. Building on this result, Knight et al. [15] conducted a study of diverse coaching populations in Canada, focusing on different stressors surrounding the coach. The findings showed that, depending on salary, level of education, agreed evaluating criteria, number of working hours and level of social support, coaches perceived their role as more or less stressful. The insight that coaches experience diverse stressors depending on, among other things, level of coaching, education and paid/non paid coaching work, highlights the need to further explore different aspects of stress in specific coaching populations.

An especially under-researched group is that of volunteer coaches in child and youth sport [13]. These coaches work under different circumstances to professional coaches, often in non-profit organizations and often being a parent of one of the children in the group, which connects the coaching work to family life [16,17]. A study by the Swedish Sports Confederation [18] showed that 80% of parents were involved in their own children’s sports club. According to Wiersma and Sherman [19] and Leberman and LaVoi [20], having a limited amount of time to volunteer, especially when having children of one’s own, was described as stressful by coaches. Solstad et al. [21] highlighted pressure on coaches from parents as stressful, and Piper et al. [22] stressed how a ‘no touch’ culture can negatively affect coach recruitment, effectiveness and relationships. In addition, Griffo et al. [23] discovered a knowledge gap regarding gender in a review on coaching literature. Previous research from the 1980s and 1990s showed that female coaches experienced more stress than male coaches [24,25,26]. Later research by Burrows [27], however, found no significant difference between men and women in perceived stress among soccer coaches in the female soccer league. Research by Leberman and LaVoi [20] and Bruening and Dixon [28] show that female coaches need to juggle work, coaching and family/domestic work and Norman’s [29] research suggests that female coaches often feel the need to prove themselves in the patriarchal world of sports, which may result in a stressful coaching situation. In light of the knowledge that women’s and men’s experiences of being a sports coach may differ, there is still limited knowledge concerning if and how gender may affect the experience of stress in sport. This makes it important to specifically explore stress in child and youth coaching, in both male and female coaches. In a study of stressors and coping among volunteer, part-time and full-time sports coaches, Potts et al. [13] found that common stressors for all three groups of coaches were athlete-related, coach-related and organizational-related. Coping strategies such as social support, time for reflection, and mentorship were central, and it was found that “the art of coping comes with experience, knowledge and understanding” (p. 64). Lastly, most research on stress among coaches is conducted within the psychological discipline, and we underline the importance of also exploring stress from a socio-pedagogical perspective. We therefore find it relevant to study stress among voluntary female and male soccer coaches in the socio-pedagogical context of youth community sports.

The overall aim of this study was to explore perceptions of stress among Swedish volunteer youth soccer coaches. The specific aims were to explore perceptions of stress in relation to; (a) personal characteristics (gender, age, ethnicity, educational level, occupation); (b) coach specific characteristics (team gender distribution, experience, own children in the team, other coaching assignments, level of coaching qualification); (c) coach specific stressors (directly and indirectly impacting the coach’s assignment).

## 2. Theoretical Framework

In this study stress is viewed as a process involving transactions between stimulus from the environment and the coach. A combination of the nature of the demands that a coach meets (stressors), the coach’s coping resources, as well as personal characteristics and the surrounding context, will influence how a coach appraises a stimulus [14]. Stressors are demands that coaches meet in the coaching environment and how a stressor is perceived depends on how the coach assesses his/her resources to cope with the stressor. Depending on the individual’s appraisal of their emotion in relation to the performance at hand, the coach will experience either a positive or a negative emotional state. The coping with this state will lead to either a positive or a negative outcome [15]. Coaches’ perception of stressors and how they value and respond to these stressors may be influenced by several different aspects, both personal and contextual. To understand how these aspects influence coaches’ perception of stress we chose to use McLeroy et al.’s [30] five levels of influence: intrapersonal, interpersonal, institutional, community and policy, to make sense of diverse aspects that may act as stressors in the coaching environment of youth soccer coaches. On an intrapersonal level, the individual coach’s perception of stress can be analyzed in relation to personal factors, such as age, sex, and level of education, and to sport coach specific attributes, such as experience of coaching (levels of coaching or years in the coaching role). The interpersonal level involves factors such as social support in the nearby environment, and also factors such as interaction with co-coaches, athletes and parents. At the institutional level the coach’s perception of stress may be influenced by formal or informal rules in the club or the type of coaching work that is expected by the club. Some examples are the type of employment the club offers (being a full or a part time coach or a volunteer coach), the clarity of the coaching mission and whether the coach is a paid or a non-paid coach, and also by factors such as work tasks within the club. Community factors can involve the local or national federation and the education that they provide. Finally, public policy relates to laws and policies such as directions from the Swedish Sport Confederation, or the national or local governing bodies affecting the coach’s work.

## 3. Materials and Methods

The data was collected through an online questionnaire which aimed to investigate experiences of coaching, knowledge, and stress among Swedish volunteer youth soccer coaches. The questionnaire contained 68 multiple-choice questions and two open-ended questions, based on McLeroy et al.’s [30] five levels of influence: intrapersonal, interpersonal, institutional, community and policy, and previous research on coaching and stress.

### 3.1. Participants

The paper is based on an online questionnaire to Swedish youth soccer coaches (coaching players 6–18 years of age). The coaches were selected through systematic sampling, which was conducted in the following steps:

- The starting point was a homepage where most of the Swedish sports clubs are registered (www.idrottonline.se).
- From the homepage every tenth club from the Swedish Football Association’s 21 regional associations were selected; the first club was randomly chosen from the list and then every tenth was chosen.
- The head coach of every team in the club was invited to participate, hence more than one coach could represent the club. Women were underrepresented as head coaches, so if the team had a female coach she was selected (even though she was not the head coach).

The questionnaire was sent in an email linking to the database of the Education Survey Automation Suite (EvaSys) on-line system. Coaches were informed about the study and its research ethics in accordance with Swedish regulations (SFS, 2008), stating that participation is voluntary, participants are free to withdraw at any time and that no unauthorized persons have access do the data (confidentiality). In total 1514 coaches were asked to participate and, after a reminder, in total 764 coaches responded to the online questionnaire (50.5% of the sample, 78.5% men and 21.5% women).

In order to ensure that only coaches during non-profit work were included in the study, participants were asked if they received any financial compensation from the club for their coaching assignment (answering options “Yes” or “No”). A small proportion (9%, n = 64) were receiving financial support from the club and consequently were omitted from further analysis. Moreover, in order to ensure that only leaders with youth coaching assignments (i.e., responsible for training and development of a youth team) were included, they were asked about their main job as a leader, responding either “Coach”, “Administration” or “Other leader work”. Of these, it turned out that seven did not have any coaching work. In addition to this, five participants did not answer this question, consequently these were also excluded from further analyses. In total 688 respondents (78% men and 22% women; 4% < 30 years, 34% between 31–40 years; 57% between 41–50 years; 5% > 51 years) with non-profit positions were included in the final analyses.

### 3.2. Measurements

In order to increase the reliability, validity and practicability of the questionnaire [31], a pilot study with ten voluntarily based male youth soccer coaches was conducted, where, among other questions (e.g., the input of economic support from the club), questions concerning how stress can be perceived and which aspects of their assignment as a coach they found most stressful were discussed, forming the basis for the final set of questions.

Personal and coach characteristics were assessed using dichotomous questions/statements (e.g., “I have my own child/children on the team”; “I receive financial compensation for my leadership assignment [i.e., salary, fee, loss of income, travel allowance or similar], multiple choice questions (e.g.,” I have completed the following coaching programs”) as well as ratio data questions (e.g., “I estimate that I spend ____ hours on average per week on my coaching assignments during the soccer season (e.g., planning and executing training and matches, travel to and from training and matches, conversations and meetings with other leaders, parents, sales activities)).

Participants’ perceived stress during the season was assessed by asking two questions (i.e., “Do you feel stressed and/or pressured in your coaching role during the ongoing soccer season?”; “Do you ever think about quitting as a soccer coach because of the stress and pressure the leadership work entails?”) forming a perceived coach stress index (α = 0.78). Participants responded on a 5-point scale, ranging from 1= No, never; 2 = Yes, once in a while; 3 = Yes, every month; 4 = Yes, every week; 5 = Yes, every day. In order to make an appropriate interpretation of the level of stress participants experienced, three groups were created (low: 1 ≤ 2.33; medium: < 2.33 ≤ 3.66; high = < 3.66 ≤ 5) using a cut point of 1.33 for each group based on the minimum and maximum value of the coach stress index.

In order to detect to what extent specific stressful factors/stressors may cause coaches to feel stressed, they were asked to respond to the question: “To what extent do the listed factors cause stress and/or pressure in your work as a coach during the soccer season?”, followed by 17 related items (e.g., “not catching up with the family enough because of the coaching”, “problems with relationships and conflicts with parents”, “having inadequate coaching skills”). Participants responded on a 5-point Likert scale, ranging from 1 = “Not at all stressful” to 5 = “Very stressful”.

### 3.3. Data Analysis

Descriptive analyses were conducted in IBM SPSS Statistics (version 25). A series of t-tests and one-way-ANOVA:s (followed by Scheffe’s post hoc tests), were used in order to test any potential differences in personal and coach characteristics in relation to perceived coach stress. T-tests were performed to detect potentially significant differences related to gender, ethnicity, own children in team, and other coaching assignments, while one-way-ANOVA:s were used to detect potentially significant differences related to age categories, highest level of education, occupation, team distribution according to sex and age, number of years as a coach, highest coach education level, number of training/competition sessions and hours per week (see Table 1). Before analysis, data was screened for normality. Values for asymmetry and kurtosis between −2 and +2 were considered acceptable in order to prove normal univariate distribution [31]. All the variables examined met the normality criteria. In order to explore which of the listed stressors are the strongest predictors of perceived coach stress, a multiple regression analysis was conducted. Before analysis, data was screened for outliers, normality, multicollinearity, and homoscedasticity of residuals. All data met the criteria and consequently were included in the analysis. The significance level for all analysis was set to 0.05. In order to interpret the meaningfulness of the results (i.e., quantifying the difference between groups) effect sizes were calculated using Cohen’s [32] criteria for estimating the effect sizes (i.e., small = 0.2, medium = 0.5, and large = 0.8 for *t*-tests (Cohen’s D/d) and one-way- ANOVA (Eta-squared/η^2^) and small ≤ 0.02, medium ≤ 0.15, and large ≤ 0.35 for stepwise multiple regression (standardized coefficient/β)).

## 4. Results

### 4.1. Characteristics of the Participants

The characteristics of the participants displayed in Table 1 show that the gender distribution of youth sport coaches in Sweden is heavily male dominant. Moreover, the majority of active coaches are in middle-age, while only a small proportion of the coaches are under 30 or over 50. An overwhelming majority of the coaches were born in Sweden, and their highest level of education is either upper secondary or university. In addition, the vast majority are on a permanent contract.

Regarding coach specific characteristics, most of the participants were coaching boys (379) or girls (262), while only a small proportion were coaching mixed teams, or both boys and girls teams at the same time. In terms of team age distribution, most of the coaches were involved in teams with youths between the ages of 10 and 15, while only a small proportion of coaches were responsible for teams with youths over 16. The vast majority have one or more of their children in their team, and almost a third of them have other coaching assignments (e.g., coaching another child’s team, being a board member in the club). Regarding participants’ highest coaching qualification, most of them have reached the third (basic) level out of four. Finally, the largest proportion of coaches are coaching 3–4 sessions/games per week, with most of them setting aside ≤1–10 h per week.

### 4.2. Level of Perceived Coach Stress

Results related to perceived coach stress (see Table 1) shows that participants in general do not feel excessively stressed being a volunteer youth sport coach (M = 2.20; SD = 0.93).

#### 4.2.1. Personal Characteristics and Perceived Coach Stress

When comparing the perceived stress level related to personal characteristics of the participants, results showed no significant differences based on gender, age, ethnicity, educational level or occupation (see Table 1). Consequently, these variables were excluded from further analyses.

#### 4.2.2. Coach Specific Characteristics and Perceived Coach Stress

When coach specific characteristics were related to perceived coach stress (see Table 1) results revealed that no differences in perceived stress could be detected in relation to team gender distribution, number of years as a coach, own children in the team, other coaching/leadership assignments or level of coaching qualification. However, results showed that coaches involved in teams with young people under 10 perceive significantly less stress than coaches involved with teams with older youths (i.e., 10 to 18 years of age). Moreover, coaches running fewer than three sessions a week feel less stressed than the other coaches. In an equal manner, coaches who spend less than 11 h and more than 20 h per week coaching their team feel less stressed.

#### 4.2.3. Coach Specific Stressors

Regarding coach specific stressors descriptive statistics (see Table 2) show that the top three reasons why youth sport coaches felt stressed are due to: (1) demands from employment, (2) difficulties catching up with the family, and (3) not having enough time to plan activities. Multiple regression analysis showed that these stressors, along with feeling pressured when selecting the team, and demands on oneself to achieve good results, significantly contributed to perceptions of stress among the investigated youth sport coaches (F = 21.75; *p* < 0.001, adjusted R^2^ = 0.34; see Table 3). It is noteworthy that perceived demands from the club and/or parents to achieve good results did not significantly predict stress among participants. Neither were coaches stressed about relations with other coaches of the team or relationships and conflicts with players in general.

## 5. Discussion

This study aimed to explore perceptions of stress in volunteer youth soccer coaches in relation to personal characteristics; coach specific characteristics; and coach specific stressors. In general, coaches perceived low levels of stress. There were no significant differences regarding perceived stress in relation to gender, ethnicity, time as a coach and coaching one’s own children. The results will be discussed in detail below based on McLeroy et al.’s [30] five levels of influence: intrapersonal, interpersonal, institutional, community and policy.

From an intrapersonal perspective there were no significant differences in stress from a gender perspective. Previous studies show different results in connection to gender. While the findings in the current study are in line with Burrows [27], more recent studies by Leberman and DaVoi [20], Norman [29] and Bruening and Dixon [28] suggest that female coaches perceive more stress in relation to factors such as juggling work, coaching and family and being part of a patriarchal coaching context. In the present study volunteer coaches in children’s and youth soccer were included; the conditions are similar for men and women within soccer, and Swedish society is also quite gender equal in general, all of which could explain why there are no significant gender differences. However, as Griffo et al. [23] state, there is need for more research on how gender affects conditions for being a coach. In former studies, coaching your own child [19,20], and not having enough qualifications [13] were considered stressful. Notably, feeling stressed when having one’s own child in the team was not seen in the current study, even though 93% coached their own child. The coaches were to some extent stressed about their qualifications. However, when considering time as a coach, most participants had coached for at least four years and thus had experience, which Potts et al. [13] found to be a good foundation for developing coping strategies. Coaching younger children and having fewer sessions per week was less stressful, which is in line with Fletcher and Scott [14]. Coaching older players was in general more stressful, meaning more focus on results and more time spent coaching, but there were only small differences. Coaches with 4–6 years as a coach, 3–4 sessions and 11–20 h per week reported more stress than the other groups of coaches. Feelings of stress when selecting the team and demands on oneself to achieve results were considered stressful. For those reasons it could be argued that coaches and players are in a phase of transition, leaving a mainly playful activity for a more competitive one, in which the level of stress increases along with increased demands to perform well. Further research is needed to clarify this assumption.

On interpersonal and institutional levels, the coaches perceived low stress regarding expectation of good results from the club and parents, and from questions concerning, for example, team selection. For instance, the study by Solstad et al. [21] showed that pressure from parents was stressful for coaches, which was not the case in this study. Since most participants in the current study are coaching young soccer players, expectation for results may not be as pronounced yet, which is in line with Fletcher and Scott [14]. In general coaches were not stressed about relations with other coaches of the team and about relationships and conflicts with players in general. Moreover, clubs have different demands and expectations on coaches to deliver quality in practice, games and other events for the players, and coaches feel stressed about not having enough time to plan these activities. They also expressed difficulties catching up with their family as stressful. These factors are connected to the stress of having limited amounts of time to volunteer, as highlighted by Leberman and LaVoi [20], and also the close connection between coaching and family life [16,17].

On the community level, demands from the coaches’ main job was considered the most stressful factor in the current study, which is also connected to the fact that the coaching is performed during leisure time [20].

When it comes to stressors that can be linked to the policy level, the coaches did not feel particularly stressed by lack of support from the club concerning how to implement policy or in terms of providing opportunities for getting qualifications. This could be explained by the fact that most of the volunteer coaches act relatively autonomously, and lead the team organization together with the other coaches without the club interfering (e.g., selecting the team and planning game tactics), which is echoed by May et al. [33].

Even though the current study targeted perceived stress among a group of coaches (i.e., volunteer coaches in children’s and youth sport), including a relatively large sample consisting of both male and female coaches, several limitations should be addressed. Overall, fewer women and participants with other ethnicity and lower general education background than the majority of the population in Sweden were included in the study. This means that the participants do not represent the population at large, but the participants reflect the composition of volunteer coaches within youth soccer in Sweden. In this study the head coaches were the primary target group. However, in the teams with a female coach, whether she was the head coach or not, the female coach was selected. If it is assumed that being head coach implies greater stress levels, then this may have affected the results, which showed only marginal gender differences. Moreover, this study only applies to volunteer coaches who are involved in soccer. It would therefore be valuable to do a similar study involving coaches in other sports, for instance, individual, technically demanding sports such as gymnastics and figure skating, in which earlier specialization is more common. Another limitation is that we did not use validated and tested instruments when assessing perceived stress. In future studies, for instance, the Perceived Stress Scale (PSS) [31] could be used. However, this strategy enabled us to measure stress from a broader perspective.

## 6. Conclusions

In conclusion, volunteer coaches cope with their coaching relatively well from a stress perspective. Perceived stress levels connected to the coaching process were low, rising slightly as the age of the players increased. The most stressful factors were demands from the coaches’ main employment, followed by catching up with the family and limited time to plan activities. Being a volunteer means challenges which interfere with one’s main job, and stress increases with age of the players. Given the complexity of volunteer coaching, there is a need to find a balance between family life, the coaching process, and surrounding structures. The results shed light on the importance of associations and clubs developing holistic strategies when recruiting and retaining coaches and participants in other functions surrounding the team in children’s and youth soccer.

## Figures and Tables

**Table 1:** Personal and Coach Characteristics of Participants Related to Level of Perceived Coach Stress (Mean).

Characteristics					
***Personal characteristics***	***n (%)***	***M (SD)***	***t/F***	***p***	***d/η^2^***
Gender			−1.31	0.189	−0.13
Male	520 (78)	2.17 (0.94)			
Female	148 (22)	2.29 (0.90)			
Age			0.23	0.877	<0.01
≤30 y	28 (4)	2.07 (0.97)			
31–40 y	230 (34)	2.18 (0.95)			
41–50 y	387 (57)	2.21 (0.91)			
≥51 y	34 (5)	2.24 (1.05)			
Ethnicity			−0.03	0.976	<0.01
Born in Sweden	646 (95)	2.20 (0.92)			
Born abroad	32 (5)	2.20 (1.11)			
Highest level of education			2.09	0.124	0.01
Upper secondary school	362 (53)	2.18 (0.94)			
University	261 (38)	2.27 (0.93)			
Others	58 (9)	2.00 (0.83)			
Occupation			1.13	0.322	<0.01
Permanent contract	599 (88)	2.18 (0.93)			
Part-time employment/	52 (8)	2.38 (0.91)			
Others (e.g., student, job-seeker, on sick leave)	25 (4)	2.12 (0.92)			
***Coach characteristics***	***n (%)***	***M (SD)***	***t/F***	***p***	***d/η^2^***
Team distribution sex/gender			0.38	0.68	<0.01
Boys	379 (56)	2.21 (0.93)			
Girls	262 (39)	2.16 (0.93)			
Others	30 (5)	2.12 (0.87)			
Team distribution age			6.38	<.001	0.03
≤9 y ^a^	142 (25)	1.89 (0.83)		<0.01 ^a,b^, ^a–c^	
10–12 y ^b^	237 (40)	2.28 (0.96)			
13–15 y ^c^	175 (30)	2.29 (0.96)			
16–18 y ^d^	30 (5)	2.28 (0.89)			
Number of years as a coach			2.60	0.075	0.01
0–3 y ^a^	193 (29)	2.07 (0.92)			
4–6 y ^b^	247 (36)	2.26 (0.88)			
≥7 y ^c^	240 (35)	2.24 (0.98)			
Own children in team			0.34	0.733	0.05
Yes	629 (93)	2.20 (0.93)			
No	49 (7)	2.15 (1.00)			
Other coaching/leader assignments					
Yes	225 (34)	2.11 (0.91)	−1.61	0.108	−0.14
No	446 (66)	2.24 (0.94)			
Highest coach education level			2.59	0.052	0.01
None	127 (19)	2.12 (0.99)			
Novice	168 (25)	2.10 (0.86)			
Basic	349 (52)	2.30 (0.94)			
Diploma/UEFA A	24 (4)	2.02 (0.88)			
Training/comp. sessions per week			6.66	.001	0.02
1–2 sessions ^a^	113 (17)	1.91 (0.80)		<0.01 ^a,b^, <0.05 ^a–c^	
3–4 sessions ^b^	475 (70)	2.26 (0.95)			
≥5 sessionsc	93 (13)	2.24 (0.95)			
Hours per week			4.43	0.012	
≤1–10 h ^a^	409 (62)	2.12 (0.90)			
11–20 h ^b^	228 (34)	2.35 (0.97)			
/≥ 21 h ^c^	24 (4)	2.15 (0.88)			
Total	688	2.20 (0.93)			

Note: ^a–d^ Scheffe´s post-hoc tests. The total number of participants in each category may differ compared to the total, due to missing data.

**Table 2:** Associations between Perceived Coach Stress Index and Participants Perceived Level of Coach Related Stressors (1 = min, 5 = max).

	*M(SD)*	1	2	3	4	5	6	7	8	9	10	11	12	13	14	15	16	17	18
1. Perceived coach stress index	2.31 (0.59)	1																	
2. Civilian employment (work, studies, etc.)	3.25 (1.16)	0.48	1																
3. Not having enough time with family because of the coaching assignment	3.17 (1.22)	0.46	0.51	1															
4. Problems with relationships/conflicts with other coaches	2.09 (1.09)	0.21	0.16	0.27	1														
5. Problems with relationships/conflicts with parents	2.23 (1.11)	0.25	0.19	0.29	0.48	1													
6. Problems with relationships/conflicts with players	1.80 (0.90)	0.16	0.18	0.18	0.34	0.49	1												
7. Inadequate organization of the club	2.72 (1.23)	0.25	0.18	0.24	0.39	0.27	0.31	1											
8. Various work efforts and sales activities to raise money for the team/club	2.80 (1.31)	0.23	0.29	0.23	0.21	0.22	0.19	0.31	1										
9. Demands from the club to achieve good results	1.36 (0.68)	0.10	0.14	0.15	0.22	0.25	0.26	0.22	0.20	1									
10. Demands from parents to achieve good results	1.69 (0.93)	0.17	0.16	0.17	0.31	0.41	0.34	0.27	0.24	0.50	1								
11. Own demands to achieve good results	2.45 (1.11)	0.27	0.23	0.21	0.21	0.24	0.26	0.21	0.21	0.22	0.31	1							
12. Poor discipline and concentration of the players during training and matches	2.80 (1.15)	0.25	0.23	0.24	0.19	0.27	0.33	0.24	0.16	0.17	0.20	0.31	1						
13. Inappropriate training and match times	2.31 (1.14)	0.28	0.29	0.26	0.22	0.23	0.23	0.24	0.31	0.21	0.27	0.22	0.26	1					
14. Not enough time to plan activities	2.79 (1.14)	0.42	0.47	0.36	0.23	0.23	0.23	0.27	0.27	0.14	0.17	0.31	0.34	0.35	1				
15. Feeling pressured when selecting the team	1.83 (0.99)	0.27	0.15	0.22	0.33	0.35	0.28	0.26	0.20	0.25	0.30	0.30	0.20	0.29	0.27	1			
16. Other coaching assignments	1.78 (1.04)	0.18	0.27	0.32	0.21	0.21	0.21	0.18	0.28	0.24	0.17	0.18	0.11	0.20	0.25	0.29	1		
17. Perceived inadequate coaching skills	2.22 (1.04)	0.26	0.24	0.20	0.21	0.23	0.32	0.22	0.21	0.22	0.22	0.28	0.30	0.22	0.37	0.32	0.28	1	
18. Lack of support from the club (e.g., policy issues and education)	1.92 (1.06)	0.23	0.22	0.23	0.36	0.28	0.29	0.53	0.22	0.24	0.27	0.27	0.24	0.30	0.31	0.29	0.31	0.35	1

Note: All correlations are significant at the 0.01 level.

**Table 3:** Multiple Regression Analysis Showing Which of the Coach Stressors Most Predicting Perceived Stress.

	B	SE B	β	*p*
Constant	0.18	0.12	-	-
Civilian employment (work, studies, etc.)	0.20	0.03	0.24	<0.001
Not having enough time with family because of the coaching assignment	0.17	0.03	0.22	<0.001
Not enough time to plan activities	0.11	0.03	0.13	<0.001
Feeling pressured when selecting the team	0.09	0.04	0.09	0.013
Own demands to achieve good results	0.06	0.03	0.07	0.045

Note: B = Unstandardized regression coefficients; SE = Standard Errors; β = Standardized regression coefficients.
